# Blocking, Bending, and Binding: Regulation of Initiation of Chromosome Replication During the *Escherichia coli* Cell Cycle by Transcriptional Modulators That Interact With Origin DNA

**DOI:** 10.3389/fmicb.2021.732270

**Published:** 2021-09-20

**Authors:** Julia E. Grimwade, Alan C. Leonard

**Affiliations:** Microbial Genetics Laboratory, Biological Sciences Program, Department of Biomedical and Chemical Engineering and Sciences, Florida Institute of Technology, Melbourne, FL, United States

**Keywords:** replication origin, DnaA, bacterial cell cycle, SeqA, factor for inversion stimulation, integration host factor

## Abstract

Genome duplication is a critical event in the reproduction cycle of every cell. Because all daughter cells must inherit a complete genome, chromosome replication is tightly regulated, with multiple mechanisms focused on controlling when chromosome replication begins during the cell cycle. In bacteria, chromosome duplication starts when nucleoprotein complexes, termed orisomes, unwind replication origin (*oriC*) DNA and recruit proteins needed to build new replication forks. Functional orisomes comprise the conserved initiator protein, DnaA, bound to a set of high and low affinity recognition sites in *oriC*. Orisomes must be assembled each cell cycle. In *Escherichia coli*, the organism in which orisome assembly has been most thoroughly examined, the process starts with DnaA binding to high affinity sites after chromosome duplication is initiated, and orisome assembly is completed immediately before the next initiation event, when DnaA interacts with *oriC*’s lower affinity sites, coincident with origin unwinding. A host of regulators, including several transcriptional modulators, targets low affinity DnaA-*oriC* interactions, exerting their effects by DNA bending, blocking access to recognition sites, and/or facilitating binding of DnaA to both DNA and itself. In this review, we focus on orisome assembly in *E. coli*. We identify three known transcriptional modulators, SeqA, Fis (factor for inversion stimulation), and IHF (integration host factor), that are not essential for initiation, but which interact directly with *E. coli oriC* to regulate orisome assembly and replication initiation timing. These regulators function by blocking sites (SeqA) and bending *oriC* DNA (Fis and IHF) to inhibit or facilitate cooperative low affinity DnaA binding. We also examine how the growth rate regulation of Fis levels might modulate IHF and DnaA binding to *oriC* under a variety of nutritional conditions. Combined, the regulatory mechanisms mediated by transcriptional modulators help ensure that at all growth rates, bacterial chromosome replication begins once, and only once, per cell cycle.

## Introduction to Orisome Assembly

Chromosome replication in bacteria ensues from a small region termed the origin of replication (*oriC*), where the double-stranded DNA is unwound to provide the appropriate configuration for assembly of two new replication forks (Leonard and Méchali, 2013). The timing of replication initiation is tightly coupled to the cell’s growth rate, such that, once a cell has reached a critical mass (and critical amount of initiator protein), a new round of chromosome replication is triggered (Cooper and Helmstetter, 1968; Løbner-Olesen et al., 1989). Once an origin has fired, no new rounds of chromosome replication are started until the next cell division cycle (Cooper and Helmstetter, 1968). To achieve this precise “once and only once per cell cycle” initiation, origin activation must be strictly regulated (Skarstad and Katayama, 2013; Katayama, 2017). Regulation is focused on assembly of a DNA unwinding machine (termed the orisome) that must be re-made each cell cycle. In all known bacterial types, orisomes comprise multiple copies of the conserved bacterial initiator protein, DnaA (Leonard and Grimwade, 2015; Bleichert et al., 2017). DnaA is also a transcriptional regulator of multiple genes (Messer and Weigel, 2003; Washington et al., 2017), including the *dnaA* gene itself (Speck et al., 1999; Hansen and Atlung, 2018).

**Figure 1:**
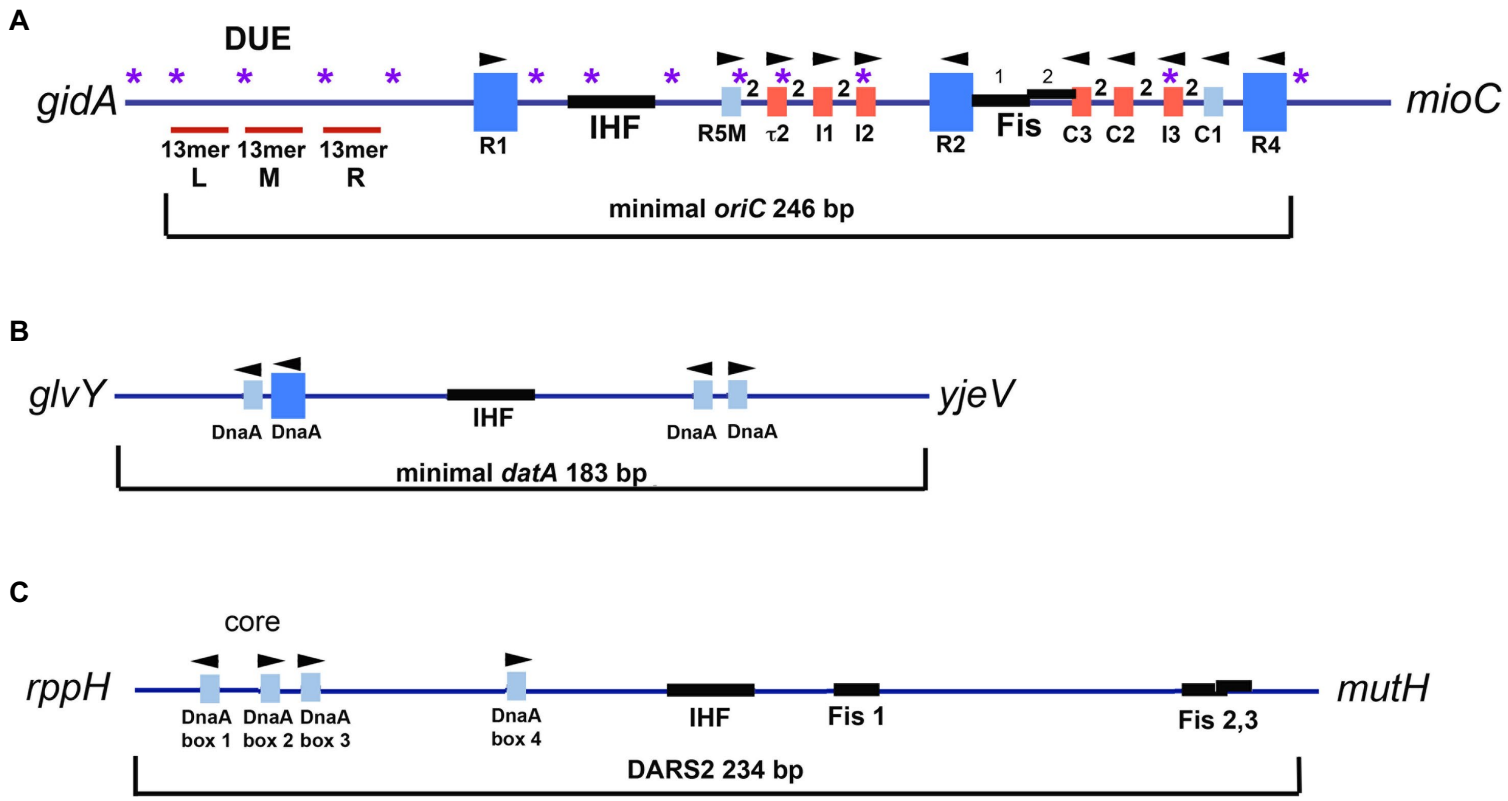
**(A)** Map of *E. coli oriC*. The large blue rectangles are high affinity DnaA recognition sites. Smaller rectangles are low affinity DnaA recognition sites that deviate from the high affinity consensus by two or more bp and are shown in red for low affinity sites that preferentially bind DnaA-ATP, and blue for low affinity sites that bind both DnaA-ADP and DnaA-ATP. The number 2 between boxes indicates the 2bp spacing between sites. The positions of the two binding sites for Fis, and the IHF binding region, are shown, as well as the left (L), middle (M), and right (R) 13mer repeats in the DNA unwinding element (DUE). Purple asterisks indicate the positions of 5′-GATC motifs, which, when hemimethylated after *oriC* is replicated, bind SeqA. Arrowheads indicate the orientation of the R285 residue in bound DnaA. The figure is adapted from Leonard and Grimwade (2015). **(B)** Map of *E. coli datA*. High and low affinity DnaA recognition sites and the IHF binding site are marked. Although the affinity of the sites has not been examined, the sites deviate from consensus by at least one bp. **(C)** Map of *E. coli* DARS2 region. DnaA recognition sites and the IHF and Fis sites are marked.

Binding of DnaA to *oriC* is cooperative (Fuller et al., 1984; Messer, 2002), and in *Escherichia coli*, DnaA binding is directed by the arrangement of specific high and low affinity nine mer recognition sites within the origin (Rozgaja et al., 2011). *E. coli oriC* contains 11 of these recognition sites (Rozgaja et al., 2011; Figure 1A), but there is tremendous diversity in the numbers and arrangements of DnaA recognition sites among bacterial origins (Zawilak-Pawlik et al., 2005; Gao et al., 2013). A detailed analysis of orisome assembly has been performed in only a few bacterial types, with *E. coli* orisomes being the most extensively studied.

**Figure 2:**
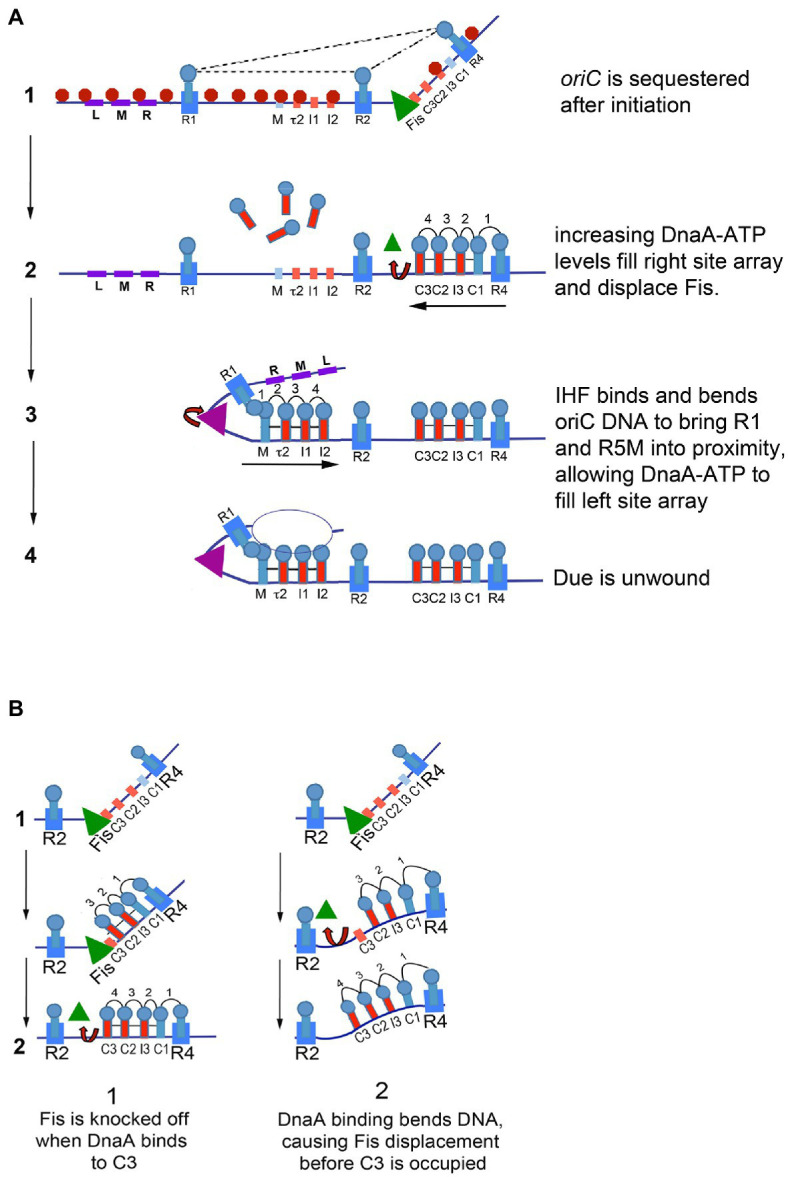
**(A)** Model of staged *E. coli* orisome assembly. Stage1: After *oriC* is replicated, SeqA (red octagons) binds to hemimethylated 5′-GATC motifs, and DnaA rebinds to high affinity R1, R2, and R4 sites. Fis (green triangle) is also bound. DnaA and Fis binding constrains the origin, possibly through interactions among the bound DnaA molecules (indicated by dotted lines), and prevents IHF from binding to its recognition site. Stage 2: DnaA bound to R4 recruits DnaA for binding to C1. DnaA-ATP then progressively fills the remaining sites in the right array. DnaA displaces Fis, and loss of Fis allows IHF to bind. Stage 3: The IHF-induced bend allows DnaA cooperative binding of DnaA between R1 and R5M. DnaA then progressively fills the remaining sites in the left array. Stage 4: The DUE is unwound and one of the single strands interacts with DnaA-ATP bound to the left array. The figure is adapted from Leonard and Grimwade (2015). **(B)** Possible mechanisms of Fis displacement by DnaA. When sufficient levels of DnaA accumulate, Fis is displaced either by direct competition with DnaA binding to C3 (left panel) or by a conformation change caused by DnaA binding to the right low affinity sites in *oriC* (right panel).

In *E. coli*, three of the eleven recognition sites (R1, R2, and R4) have the high affinity “consensus” sequence 5′-TTATNCACA (Schaper and Messer, 1995). These three sites are occupied throughout the cell cycle (Samitt et al., 1989; Cassler et al., 1995; Nievera et al., 2006) and may interact to constrain the origin (indicated by dotted lines in Figure 2A; constraint is also discussed below). The two gap regions between R1 and R2, and R2 and R4 each contain an array of four lower affinity sites, separated by two base pairs, whose sequences differ from consensus at two or more positions (Figure 1A; Leonard and Grimwade, 2010; Rozgaja et al., 2011). The lower affinity sites are occupied only at the time of initiation (Nievera et al., 2006). Completion of orisome assembly requires that the high affinity sites recruit and donate DnaA-ATP molecules to the arrays (Schaper and Messer, 1995; Miller et al., 2009; Figure 2A). Since DnaA-ADP bound to high affinity sites are capable of donation (Noguchi et al., 2015), it is likely the first interactions utilize the N-terminal domains (Domain 1). However, only the ATP-bound form of DnaA can engage in the cooperative binding needed to “fill the gaps” because only this form is recognized by six of the eight low affinity sites (tau2, I1, I2, I3, C2, and C3; McGarry et al., 2004; Grimwade et al., 2018). Domain III interactions are also required for this “gap filling” stage (Kawakami et al., 2005). Once bound to the arrayed sites, it is likely that the DnaA molecules form two pentameric oligomers in each half of *oriC* (Rozgaja et al., 2011; Shimizu et al., 2016). Although the exact structure of the pentamers is not known, it is likely that they cause the DnaA-*oriC* complex to assume a curved configuration (Shimizu et al., 2016), since DnaA binding is reported to bend DNA about 40 degrees (Schaper and Messer, 1995). It has been suggested that this DnaA-induced bending of *oriC* might create some of the stress required for origin unwinding (Grimwade et al., 2018). Consistent with this idea, immediately after the arrays are filled with DnaA-ATP, the A-T rich DNA unwinding element (DUE) at the left end of *oriC* becomes unwound and available for replicative helicase loading (Kowalski and Eddy, 1989; Bell and Kaguni, 2013; Rozgaja et al., 2011; Figure 2A). One of the single strands of DNA in the unwound region is also occupied by oligomeric DnaA-ATP, which may interact with sequences similar to the so called DnaA trio motifs found in other bacterial origins (Ozaki and Katayama, 2012; Richardson et al., 2016). Binding to single-stranded DNA has been proposed to help induce unwinding and to stabilize the unwound strands (Speck and Messer, 2001; Ozaki and Katayama, 2012). Because the ATP-bound form of DnaA is required for both double-stranded and single-stranded *oriC* binding, mechanisms that regulate cellular levels of DnaA-ATP play a key role in precise initiation timing (Skarstad and Katayama, 2013; Riber et al., 2016; Katayama et al., 2017).

Although DnaA-ATP alone is sufficient to build an orisome capable of triggering initiation in *E. coli*, its *oriC* also contains recognition sites for three accessory proteins: SeqA (Slater et al., 1995; Waldminghaus and Skarstad, 2009), integration host factor (IHF; Nash and Robertson, 1981; Polaczek, 1990; Rice, 1997), and factor for inversion stimulation (Fis; Finkel and Johnson, 1992; Filutowicz et al., 1992; Figure 1A). Binding of these three proteins to *oriC* regulates DnaA-ATP’s occupation of lower affinity recognition sites (Grimwade et al., 2000; Ryan et al., 2004). At other chromosomal loci, SeqA, IHF, and Fis function as transcriptional modulators and may also be involved in organizing the bacterial chromosome (Schmid, 1990; Dorman and Deighan, 2003; Løbner-Olesen et al., 2003; Klungsøyr and Skarstad, 2004; Seshasayee et al., 2011; Dorman, 2014; Wang and Maier, 2015; Hołówka and Zakrzewska-Czerwińska, 2020). Although none of these three proteins is essential for *E. coli* viability, loss of any of them results in altered host growth (Filutowicz and Roll, 1990; Filutowicz et al., 1992; Von Freiesleben et al., 2000; Waldminghaus and Skarstad, 2009). Below, we review these transcriptional modulators and describe how they are utilized during orisome assembly to impart precise regulation of replication initiation.

## SeqA, a Negative Regulator of Initiation That Blocks Low Affinity DnaA Binding Sites

Successful formation of unwound orisomes results in recruitment of the replicative helicase, followed by assembly of new replication forks which then copy *oriC* DNA and move bidirectionally toward the terminus region of the chromosome. These processes remove DnaA from *oriC* but do not immediately reduce the amount of DnaA-ATP available to rebind to the origin, which, if it occurred, would result in re-initiation and over-replication. Seminal studies by Messer and Russell and Zinder (Messer et al., 1985; Russell and Zinder, 1987), examining *in vivo* replication of minichromosomes (plasmids initiating replication from a cloned copy of *oriC*) in strains deficient in DNA methylation, identified a role for DNA hemimethylation in the prevention of re-initiation. When DNA is replicated, the older strand carries methyl-adenines in the palindromic sequence 5′-GATC, while the daughter strand is unmethylated. This state of hemimethylation continues until DNA adenine methylase (encoded by the *dam* gene) methylates the daughter strand, restoring the parental methylation pattern (Geier and Modrich, 1979).

The *E. coli oriC* region contains an unusually high density of 5′-GATC motifs (Oka et al., 1984; sites are marked in Figure 1A); the motifs are found throughout the DUE, as well as in the IHF binding region and inside or overlapping several low affinity DnaA recognition sites (R5M, tau2, I2, and I3). After passage of the replication fork, these 5′-GATC sequences in *E. coli oriC* remain hemimethylated for approximately 1/3 of the cell cycle (Campbell and Kleckner, 1990) because the SeqA protein “sequesters” the origin from the *dam* methyltransferase (Lu et al., 1994; Figure 2A). SeqA is also a transcriptional regulator, rendering some promoter regions, including those for *dnaA* (Campbell and Kleckner, 1990) and *gidA*, the gene adjacent to the *oriC* DUE (Theisen et al., 1993) inactive for part of the cell cycle. There is also evidence that SeqA can be a positive regulator of transcription in bacteriophage lambda (Slomińska et al., 2001).

SeqA binds preferentially to hemimethylated 5′-GATC sequences, with a much lower affinity for the fully methylated version; it does not bind at all to unmethylated 5′-GATCs (Brendler et al., 1995; Slater et al., 1995). The basal SeqA structure is a dimer, and dimers can oligomerize to form filaments (Guarné et al., 2005). SeqA oligomers can interact with large stretches of DNA, resulting in large toroidal complexes that constrain negative supercoils (Klungsøyr and Skarstad, 2004; Skarstad and Katayama, 2013). These complexes localize at replication forks (Hiraga et al., 2000; Molina and Skarstad, 2004) and SeqA assemblies have been proposed to help organize origins and replication forks by pairing sister origins and forks in rapidly growing *E. coli*, so that they eventually segregate properly (Fossum et al., 2007; Morigen et al., 2009).

At *oriC*, SeqA prevents orisome re-assembly immediately after an initiation event (Nievera et al., 2006). SeqA occupation of *oriC*’s hemimethylated 5′-GATC sites permits DnaA to rebind higher affinity sites, resetting the origin, but blocks DnaA binding to the lower affinity sites (Nievera et al., 2006; Taghbalout et al., 2000; Figure 2A). While the mechanism of SeqA blocking is not fully understood, it seems likely that SeqA, by occupying hemimethylated 5-GATC motifs, physically obstructs DnaA’s access to the DUE and to its low affinity recognition sites. SeqA could also inhibit IHF bending, which would reduce cooperative DnaA binding in the entire left arrays of low affinity sites (IHF actions are described in more detail below). Some of the inhibition of initiation caused by SeqA may also result from a reduction in negative superhelicity (Torheim and Skarstad, 1999). Additionally, the inhibition of transcription from the *dnaA* gene by SeqA (Campbell and Kleckner, 1990; Theisen et al., 1993) contributes to the decrease in cellular DnaA-ATP levels in the cell after initiation (Kurokawa et al., 1999), so that when *oriC* becomes fully remethylated, DnaA-ATP levels are low enough so that the loss of SeqA blocking does not result in immediate re-initiation (Bogan and Helmstetter, 1997).

## Dynamic Binding of the DNA-Bending Proteins Fis and IHF to Specific Sites in *oric* Regulates Ordered Orisome Assembly

Unlike SeqA, which was identified in a screen for regulators of initiation, both Fis and IHF were originally identified as players in phage recombination and subsequently were discovered to participate in many cellular DNA transactions including replication initiation (described below), transcription, recombination, and transposition, see reviews (Friedman, 1988; Finkel and Johnson, 1992). Both proteins contribute to regulated expression of hundreds of *E. coli* genes, reviewed in (Martínez-Antonio and Collado-Vides, 2003). Depending on the position of recognition sites within promoters, Fis and IHF can be either repressors or activators and there are many genes whose promoters contain recognition sites for both factors (Monteiro et al., 2020). A key feature of both proteins is their ability to bend DNA to an impressive degree; Fis may bend its target by 60 to 90 degrees (Gille et al., 1991) and IHF can produce 180 degree hairpins (Friedman, 1988; Ellenberger and Landy, 1997; Rice, 1997; Travers, 1997; Engelhorn and Geiselmann, 1998). Bending by these proteins also imparts topological effects on supercoiled DNA and plays a role in setting the degree of supercoiling in nucleoids (Schneider et al., 1999; Travers et al., 2001; Opel et al., 2004).

In *E. coli*, regulation of replication initiation by Fis and IHF requires binding of the two proteins to specific recognition sites within *oriC* (Roth et al., 1994). Two closely spaced sites for Fis have been identified between the high affinity R2 site and the array of low affinity sites in the right half of the origin (C3-C1; Figure 1A; Gille et al., 1991; Filutowicz et al., 1992; Hengen et al., 1997). One of the Fis sites partially overlaps the C3 site, which may have ramifications with regard to initiation regulation (described below). The binding site for IHF is in the left region of *oriC*, between the high affinity R1 site and the left array of low affinity sites (Polaczek, 1990; Figure 1A).

The roles of IHF and Fis in initiation regulation are not as straightforward as that of SeqA. IHF was shown to be a positive regulator in the *in vitro* replication and unwinding of minichromosomes (Hwang and Kornberg, 1992), as well as in the replication of some other plasmid systems (Filutowicz and Appelt, 1988; Biek and Cohen, 1989). In contrast, studies examining the role of Fis in *E. coli* replication initiation report both positive and negative effects (Hiasa and Marians, 1994; Roth et al., 1994; Wold et al., 1996; Margulies and Kaguni, 1998; Flåtten and Skarstad, 2013). Greater insight was provided by examining the binding of Fis and IHF during the cell cycle (Cassler et al., 1995; Ryan et al., 2002).

During the cell cycle, Fis and IHF bind to *oriC* at different times (Cassler et al., 1995). Fis occupies its recognition site most of the cell cycle but is not bound during the time of replication initiation; in contrast, IHF interacts with its *oriC* site only transiently, immediately before the DNA unwinding step and when Fis is not bound (Figure 2). These results provided evidence that the two proteins play opposing roles in initiation, with the simplest scenario being that Fis acts as an inhibitor of orisome formation, while IHF is stimulatory.

The positive effect of IHF relies on the bending of *oriC* DNA, which facilitates DnaA cooperative binding (Grimwade et al., 2000). IHF, when bound to its recognition site between R1 and R5M, stabilizes a bend in *oriC* that puts R1 in proximity to R5M, allowing DnaA bound at R1 to recruit additional DnaA and donate it to R5M (Figure 2); DnaA-ATP binding can then extend to all the arrayed sites (Leonard and Grimwade, 2010; Rozgaja et al., 2011). Additionally, the IHF-induced bend places the DUE in *E. coli oriC* near the low affinity sites in the left half of the origin, which permits interaction between the array-bound DnaA-ATP and single-stranded DNA in the DUE (Ozaki and Katayama, 2012; Ozaki et al., 2012; Sakiyama et al., 2017; Figure 2).

Cellular levels of IHF should be high enough to allow it to bind to *oriC* throughout the cell cycle. However, the finding that IHF binding is restricted to the time of initiation (Cassler et al., 1995) suggested that some factor prevented the interaction of IHF with its *oriC* recognition site. Examination of orisome formation *in vitro* revealed this inhibitory factor was Fis, even though the Fis and IHF recognition sites are separated by nearly 90bp (Ryan et al., 2004). Fis inhibition begins in the early stages of orisome assembly, when *oriC* is sequestered by SeqA (described above). At this time, the origin is bound to Fis as well as to DnaA at R1, R2, and R4 (Figure 2A). Binding of Fis and DnaA topologically constrains *oriC*, and the constraint is sufficient to prohibit binding of IHF at physiological concentrations (Kaur et al., 2014; Figure 2). Although the details of the configuration of the constrained complex remain incomplete, there are data that suggest it is maintained by interactions among DnaA molecules bound to R1, R2, and R4 (Kaur et al., 2014). Whatever its nature, the constraint imparted by DnaA and Fis blocks IHF binding until increasing concentrations of DnaA displace Fis shortly before initiation (Ryan et al., 2004; Figure 2B). Once Fis leaves *oriC*, IHF binding and bending rapidly promotes DnaA binding and DUE unwinding (described above). Thus, the dynamic binding of Fis and IHF during the cell cycle establishes an initiation “on switch” (Ryan et al., 2004).

Key to the Fis-IHF initiation switch is the interplay between Fis and DnaA-ATP, mediated by the position of the Fis binding site at the end of the right array of low affinity. In the presence of Fis, only the right half of *oriC* can direct cooperative DnaA binding, since the interaction of DnaA at R1 and R5M in *oriC*’s left half is prohibited until IHF binding and bending takes place (Rozgaja et al., 2011; Figure 2A). Growth of the right DnaA pentamer competes with Fis binding, and initially Fis wins the contest and delays DnaA-ATP binding (Ryan et al., 2004). However, as cellular DnaA-ATP levels rise, DnaA displaces Fis while simultaneously completing assembly of the DnaA pentamer associated with the right array of sites (Ryan et al., 2004). It is not entirely clear how DnaA displaces Fis, but there are two possible scenarios (Figure 2B). The first is that Fis is simply knockedoff its site when the DnaA oligomer extends to C3. The second possibility is that a DnaA-induced curve in *oriC* DNA (Schaper and Messer, 1995; Shimizu et al., 2016) weakens Fis interactions enough for Fis to be released from *oriC* before DnaA occupation of the right site array is complete.

## The Fis-IHF Switch Also Regulates Initiation Synchrony in Rapid Growth

Members of the *Enterobacteriaceae* (including *E. coli*) and some other bacterial types are capable of rapid growth (doubling their numbers every 20min) under nutrient-rich conditions, for example, see (Schaechter et al., 1958). However, except in very slow growing cells, the time to replicate the chromosome is constant over a range of growth rates, measured to be around 40–50min in a variety of *E. coli* strains (Cooper and Helmstetter, 1968; Helmstetter et al., 1992). If the time required to replicate the genome exceeds the generation time, new initiations from *oriC* must take place before previous rounds of chromosome replication are completed (Cooper and Helmstetter, 1968). Thus, bacteria growing rapidly in rich media contain multiple copies of *oriC*, all of which fire synchronously (Skarstad et al., 1986). Synchronous initiation is observed even when the cell contains extra copies of *oriC* carried on minichromosomes (Helmstetter and Leonard, 1987), indicating that the initiation mechanism does not “count” the number *oriC* copies. Since assembly of each orisome requires the same amount of new DnaA-ATP, and mass at initiation is fairly constant regardless of how many origins the cell contains (Donachie, 1968), the attainment of initiation synchrony presents a bit of a puzzle. One model suggests that there is an initiation cascade that resembles a chain reaction, where the DnaA that is displaced when one copy of initiating *oriC* is rapidly picked up and used to complete assembly of another orisome (Løbner-Olesen et al., 1994). However, even a cascade would result in some asynchrony, suggesting that there could be additional regulation to ensure that all origins fire at the same time.

**Figure 3:**
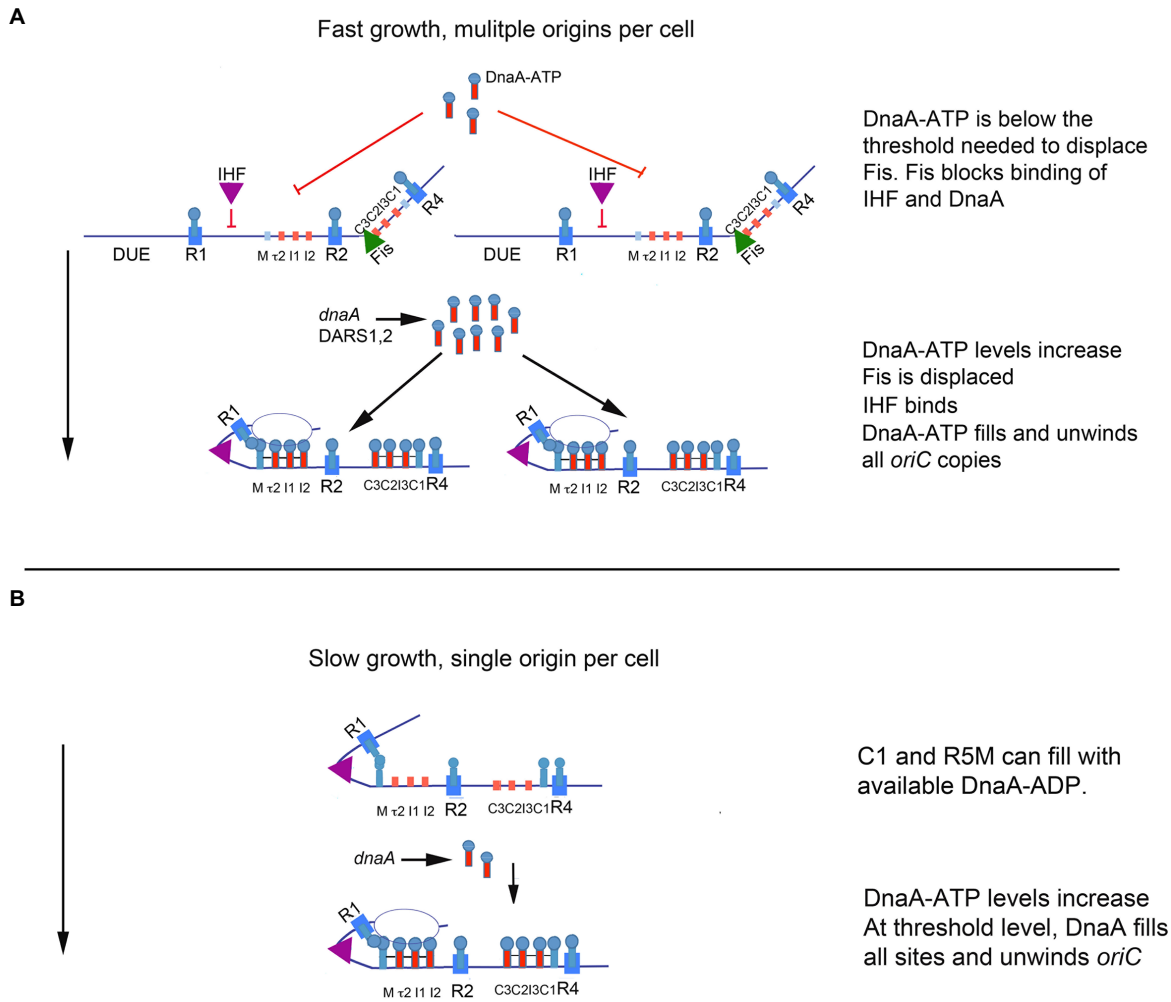
Model of orisome assembly in fast and slow growing cells. **(A)** In rapidly growing cells, DnaA-ATP site occupation is regulated by Fis. Bound Fis prevents free DnaA-ATP from occupying *oriC* and excess DnaA-ATP (provided by new synthesis and DARS2) accumulates. When sufficient DnaA-ATP becomes available, Fis is displaced. Loss of Fis allows IHF to bind to *oriC*, and available DnaA-ATP binds to low affinity sites on all *oriC* copies in the cell. Orisome assembly may be completed by an initiation cascade (see text for details). **(B)** In slowly growing cells, cells contain a single copy of *oriC*, bound to IHF throughout the cell cycle. All low affinity DnaA-ATP sites become occupied as DnaA-ATP becomes available (see text for details). When enough DnaA-ATP has accumulated to fill all sites in the left region of *oriC*, the origin is unwound. The figure is adapted from Rao et al. (2018).

The switch mechanism imparted by the dynamic interactions of Fis and IHF, described above, could provide the regulation that fine tunes an initiation cascade, if the amount of DnaA required to displace Fis during the cell cycle exceeded the amount required to fill the remaining empty DnaA binding sites on other origin copies (Figure 3A). In this scenario, the cell would contain more DnaA than needed to complete orisome assembly at any individual *oriC* copy, so loss of Fis would cause both the rapid binding of IHF and provide available DnaA for unfired *oriC* copies (Ryan et al., 2004). Initiation and the resulting orisome disassembly would further increase the amount of available DnaA-ATP, which could cascade to any remaining unfired origins (Figure 3A). Evidence for both Fis and IHF acting as modulators of initiation synchrony is shown by the asynchronous initiations observed in fast-growing *E. coli* cells carrying loss-of-function mutations in the genes encoding Fis or IHF (Riber et al., 2016). Further, rapidly growing Fis null cells not only have asynchronous initiations, they also have fewer origins than wild-type cells (i.e., the Fis null cells under-initiate; Flåtten and Skarstad, 2013; Rao et al., 2018), consistent with the idea that, without Fis, there is not enough DnaA-ATP in the cell for every orisome to complete its assembly. In other words, by being an inhibitor of orisome assembly, Fis becomes a positive regulator of initiation synchrony.

## Growth Regulation of Fis Produces Different Orisome Assembly Paths for Fast Growth, Slow Growth, and Stationary Phase

The regulatory role of Fis in orisome assembly (described above) must be limited to the more rapid growth rates of cells containing multiple copies of *oriC*, because Fis expression is growth rate regulated, with high levels (10–15 thousand molecules per cell) maintained during rapid growth and low to no protein (less than 100 molecules per cell) present during slow growth and stationary phase (Ball et al., 1992; Schneider et al., 1997). Since IHF is not growth rate regulated (Ditto et al., 1994), the absence of Fis in slowly growing cells should result in a lack of Fis inhibition of IHF binding to *oriC*. If so, this should cause orisome assembly to begin with R1 and R5M already in proximity due to bound IHF, and the low affinity site arrays in both left and right regions of *oriC* should start filling with DnaA at approximately the same time (Figure 3B). However, the left region of *oriC* has been shown to be more important for unwinding (Ozaki et al., 2012) and it is possible that the right region has little function during slow growth. This idea is consistent with previous findings that some deletions in the right half of *oriC* cause origin inactivation under fast growth, but not slow growth conditions (Stepankiw et al., 2009).

The absence of Fis during slow growth also prevents the cell from accumulating any more DnaA-ATP than is required to assemble an orisome on its single copy of *oriC*, and it is likely that sites in *oriC* become occupied as soon as levels of free DnaA-ATP are high enough to bind to them. Interestingly, recent studies revealed that in slow growth conditions, the tau2 site in the left half of *oriC* may represent a bottleneck in orisome assembly (Rao et al., 2018), determining how much DnaA-ATP is needed. The reason for this is not known, but it may be related to tau2’s position as the first low affinity DnaA-ATP site in the array (Figure 1A); R5M binds both nucleotide forms of the initiator equally and is likely to be occupied by DnaA-ADP *in vivo* (Grimwade et al., 2007). Logically, slow growth orisomes could start orisome assembly by using available DnaA-ADP for cooperative binding to R5M but would have to pause until levels of DnaA-ATP were high enough to extend binding to tau2.

At intermediate growth rates, cells contain lower but detectable levels of Fis. This creates the interesting, although speculative possibility that depending on growth rate, Fis may occupy one or both of its two recognition sites between R1 and C3, producing different levels of repression over a range of growth conditions (Gille et al., 1991; Hengen et al., 1997, 2003). If this is the case, then a reasonable corollary would be that as Fis repression decreases, the number of DnaA-ATP molecules required to build an unwound orisome would also decrease. Consistent with this idea, there is evidence in the literature that initiation timing is regulated differently in fast and slow growing cells (Wold et al., 1994; Zheng et al., 2020). It is also possible that regulators of DnaA-ATP regeneration (mediated by DARS2 locus, see below) may function differently during fast and slow growth in ways that could affect initiation timing.

The behavior of Fis and IHF also changes at *oriC* as fast-growing bacteria enter stationary phase, stop initiating chromosome replication, and return to the single copy state (Boye and Løbner-Olesen, 1991). Since the levels of IHF increase and the Fis levels become very low, the stationary phase *oriC* is complexed with IHF (Ditto et al., 1994; Cassler et al., 1999). Unless an undiscovered stationary phase repressor of initiation also exists, it must be assumed that these orisomes are incompletely assembled due to insufficient DnaA. This configuration may be advantageous for cells to restart growth when conditions allow, although the effect will be transient, since chromosomal origins that leave stationary phase for nutritionally rich conditions will rebind Fis as cells enter early exponential growth phase, when Fis levels peak (Ball et al., 1992). It is also possible that IHF binding to *oriC* helps the cell in other ways, perhaps by setting *oriC* in a configuration that is beneficial for long-term non-initiating conditions followed by a stepwise origin reset.

### SeqA, Fis, and IHF Modulate Levels and Activity of Other Initiation Regulators

In addition to their participation in orisome assembly, SeqA, Fis, and IHF also indirectly affect initiation timing by interacting with non-*oriC* sites on the *E. coli* chromosome that help regulate intracellular DnaA-ATP levels (Katayama et al., 2010; Leonard and Grimwade, 2015; Riber et al., 2016).

Following each new start of chromosome replication, DnaA-ATP levels in cells decrease (Kurokawa et al., 1999). In part, this is because SeqA sequesters the *dnaA* gene region and prevents new DnaA-ATP synthesis for approximately 20% of the cell cycle (Campbell and Kleckner, 1990). Although the mechanism by which SeqA blocks *dnaA* transcription gene has not been thoroughly explored, there is a high density of 5′-GATC residues in the *dnaA* gene region, similar to that found in *oriC*, which, when hemimethylated, could support a SeqA complex that prevents binding of transcriptional activators. Additionally, a replisome-associated protein (Hda) stimulates the ATP hydrolysis of any DNA-bound DnaA that it encounters (Kato and Katayama, 2001). While this mechanism, termed regulatory inactivation of DnaA (RIDA), is the primary means to inactivate DnaA-ATP, a secondary inactivation mechanism exists for any unbound DnaA-ATP that remains after initiation (for example, from DnaA-ATP oligomers displaced from a newly replicated origin). Secondary inactivation is dependent on a DNA locus termed *datA* (about 47kbp from *oriC*), whose deletion causes intracellular levels of DnaA-ATP to increase (Ogawa et al., 2002; Kasho and Katayama, 2013). *DatA* contains an essential binding site for IHF and two sets of paired DnaA recognition sites (Kasho and Katayama, 2013; Figure 1B). The binding of IHF at *datA*, like *oriC*, is dynamically regulated during the cell cycle by mechanisms that are not yet known, but IHF binding at *datA* is a post-initiation event (detected for 20–30min after initiation during rapid growth). IHF most likely bends the DNA to bring the two pairs of DnaA sites into proximity (Katayama et al., 2017), to promote the formation of new DnaA multimers on *datA* that are then susceptible to RIDA (Kasho et al., 2017).

A second locus regulated by Fis and IHF, termed DARS2, is located halfway between *oriC* and the chromosomal terminus. DARS2 raises the level of active DnaA *via* a mechanism that removes the nucleotide from bound DnaA-ADP to promote rapid regeneration to DnaA-ATP (Fujimitsu et al., 2009; Sugiyama et al., 2019) (An additional site, termed DARS1, also contributes to increasing DnaA-ATP levels but is not regulated by Fis or IHF). Regenerated DnaA-ATP is used during rapid growth where new synthesis is not sufficient to produce the required threshold level for Fis displacement and synchronous initiation at all origin copies (described above). DARS2 contains four DnaA recognition sites critical for regeneration activity, positioned near an essential IHF recognition site (Figure 1C). Three of these sites are closely spaced and form what is termed the DARS “core” (Figure 1C; Sugiyama et al., 2019). DARS2 activity is dependent on binding of both IHF and Fis, and similar to their dynamic binding at *oriC*, Fis binds DARS2 throughout the cell cycle, and IHF binds only to activate DARS2 immediately prior to the initiation step (Kasho et al., 2014). It is not yet known whether Fis is responsible for restricting IHF binding, as is the case for the orisome. It is also unclear how Fis and IHF promote the interactions among the three bound DnaA-ADP molecules to effect the disassociation of ADP, although it seems likely that DNA bending is involved to bring DnaA molecules together. However, since Fis is growth rate regulated, it is logical to think that the amount of DnaA-ATP made available by regeneration at DARS2 will also vary in fast and slow growing cells.

Binding of IHF to *oriC*, *datA*, and DARS2 is temporally regulated during cell cycle progression such that *oriC* is bound first, then *datA*, and then DARS2 (Kasho and Katayama, 2013; Kasho et al., 2014). While the mechanism responsible is not yet defined, the movement of the replication fork may cause the displacement of IHF from one location to another as the chromosome is replicated. This idea is supported by studies which show that the chromosomal positions of *datA* and DARS2 relative to *oriC* are important for cell cycle control and bacterial fitness (Frimodt-Møller et al., 2016; Inoue et al., 2016).

## Conclusion

The dynamic bending, blocking, and binding properties of the transcriptional regulators SeqA, Fis, and IHF provide an elegant solution to multiple problems that could disrupt properly timed initiation and eventually prevent equivalent inheritance of genomic DNA in daughter cells. One pitfall that must be avoided after every initiation event is a second triggering of the same origin, which can result in closely spaced replication forks that could collide and then collapse. SeqA helps prevent this immediately after initiation (Sutera and Lovett, 2006), by blocking DnaA’s access to *oriC* and preventing reformation of the orisome. Coincidently, SeqA inhibition of new DnaA-ATP synthesis contributes to the reduction in initiation potential (Skarstad and Katayama, 2013). IHF also safeguards against re-initiation by binding and bending the *datA* locus to promote the formation of a bound DnaA-ATP complex that then can be inactivated by RIDA.

Asynchronous initiations are also problematic to replicating *E. coli* cells. If all origins in rapidly growing cells do not fire synchronously, when cells divide, the daughter cells will contain different amounts of DNA, which could result in eventual formation of anucleate cells. The dynamic binding of Fis and IHF at *oriC* promotes initiation synchrony because it delays DnaA occupation of *oriC* until sufficient DnaA-ATP has accumulated to fill multiple origins (described above, and in Figure 3A). Further, since Fis is growth rate regulated, its effect on the amount of DnaA-ATP needed for initiation is different at different growth rates, which helps solve the problem of ensuring that orisome assembly and DnaA-ATP availability are compatible with specific cell cycle stages over a wide variety of growth rates (Figure 3B).

While transcriptional modulators clearly play a key role in regulating *E. coli* origin function during the cell cycle, far less is known about other bacterial types. There are clearly transcriptional regulators that bind to other bacterial origins and regulate initiation; these include CtrA, SpoOA, AdpA, and MtrA in *C. crescentus*, *B. subtilis*, *S. coelicolor*, and *M. tuberculosis*, respectively, reviewed in (Wolanski et al., 2014; Grimwade and Leonard, 2019). However, for the most part, how precise initiation timing is achieved in most bacterial types is not well understood. It is certainly possible that part of the immense diversity found among bacterial replication origins are caused by the need for different types of regulation, some of which could involve transcriptional modulators. Hopefully, expanding studies to greater numbers of bacterial types will determine whether analogs of SeqA, Fis, and IHF regulate initiation by similar strategies or will reveal new uses of transcriptional regulators in diverse bacterial orisome assemblies.

## Author Contributions

JG and AL contributed equally to the writing of this article.

## Funding

The work from our laboratory that is cited in this review was supported by the Public Health Service grant no. GM54042. Publication of this article was funded by the Open Access Subvention Fund of the Florida Tech Libraries and by the Office of Dean of the Florida Tech College of Engineering and Science.

## Conflict of Interest

The authors declare that this work was done in the absence of any commercial or financial relationships that could be construed as a conflict of interest.

## Publisher’s Note

All claims expressed in this article are solely those of the authors and do not necessarily represent those of their affiliated organizations, or those of the publisher, the editors and the reviewers. Any product that may be evaluated in this article, or claim that may be made by its manufacturer, is not guaranteed or endorsed by the publisher.
